# *NOD2/CARD15* gene mutation identified in a Chinese family with Blau syndrome

**Published:** 2012-03-09

**Authors:** Haotian Xiang, Ting Zhang, Mengping Chen, Xiaomin Zhou, Zhen Li, Naihong Yan, Shiguang Li, Yu Han, Qiyong Gong, Xuyang Liu

**Affiliations:** 1 Ophthalmic Laboratories & Department of Ophthalmology, Translational Neuroscience Center, West China Hospital, Sichuan University, Chengdu, P.R. China; 2 Department of Ophthalmology, the Second People's Hospital of Zhengzhou, Zhengzhou, P.R. China; 3 Department of Ophthalmology, the People's Hospital of Leshan, Leshan, P.R. China; 4 Department of Radiology, West China Hospital, Sichuan University, Chengdu, P.R. China; 5 Shenzhen Eye Hospital, Jinan University, Shenzhen, P.R. China

## Abstract

**Purpose:**

To characterize the clinical features of a Chinese pedigree with Blau syndrome and to identify mutations in the *NOD2/CARD15* (nucleotide-binding oligomerization domain containing 2/caspase recruitment domain family, member 15) gene.

**Methods:**

Clinical features of this family were evaluated. Genomic DNA was obtained from blood samples, and all exons of *NOD2/CARD15* were amplified by polymerase chain reaction (PCR) and direct DNA sequencing of PCR products was performed for mutations in *NOD2/CARD15*.

**Results:**

Granulomatous arthritis, uveitis, and skin granulomas were found in all affected members. Sequencing analysis demonstrated a heterozygous C>T mutation in exon 4 of N*OD2/CARD15* in all patients of this pedigree, which resulted in an amino acid substitution at position 334 (p.R334W).

**Conclusions:**

The R334W mutation in NOD2/CARD15 caused Blau syndrome in a Chinese pedigree. This is the first report of R334W mutation in NOD2/CARD15 in a Chinese pedigree of this disease.

## Introduction

Blau syndrome (BS, OMIM 186580) is a rare, autosomal dominant and autoinflammatory disorder with an onset under 4 years of age, which is characterized by clinical triad of symmetric arthritis, recurrent uveitis, and granulomatous dermatitis [1]. The disease was first described by Blau in 1985 [2], and then a similar family was reported by Jabs et al. [3] in the same year. Mutations in *NOD2/CARD15* (nucleotide-binding oligomerization domain containing 2/caspase recruitment domain family, member 15) were first identified to be associated with susceptibility to Crohn's disease (CD) [4], which improved our knowledge of systemic autoinflammatory disorders. Subsequently, the same gene defects in families with Blau syndrome and early onset sarcoidosis (EOS) have also been described [5,6].

The *NOD2* (nucleotide-binding oligomerization domain containing 2) gene, also termed *CARD15* (caspase recruitment domain family, member 15), is located on chromosome 16q12 and encodes multidomain protein of 1,040 amino acids. The NOD2 protein is primarily expressed in antigen-presenting cells, such as monocytes and macrophages, and in intestinal Paneth cells [7]. It plays an important role in the immune response to intracellular bacterial lipopolysaccharides (LPS) by activating the nuclear regulatory factor (NF)-kB (nuclear factor-kappa B) pathway of inflammation and apoptosis [8]. Subsequent researches of the pathogenic mechanism responsible for the chronic inflammation are still under investigation. The most common *NOD2/CARD15* mutations in Blau Syndrome were found in codon 334 (R334W and R334Q) in French, German [6], Japanese [9,10], and Italian [11] families, while other mutations have also been reported, such as T605N [12], E383K [11,13], and L469F [11].

In this study, we conducted a clinical evaluation and mutational analysis of *NOD2/CARD15* in a Chinese family with Blau syndrome. The R334W mutation of NOD2/CARD15 was identified in all affected member of this pedigree. To the best of our knowledge, this is the first report of R334W mutation in *NOD2/CARD15* in a pedigree with this disease in Asia except Japan [9,10].

## Methods

### Patients

**Figure 1:**
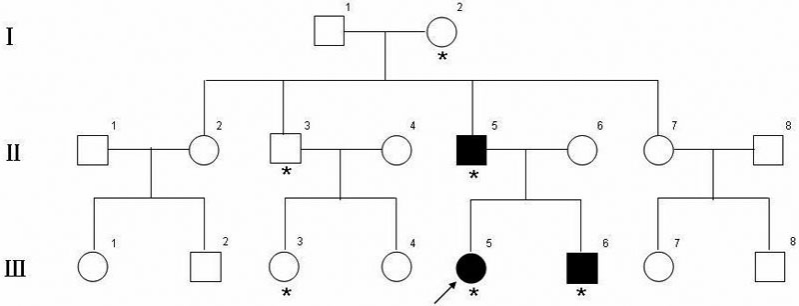
Pedigree of a Chinese family with Blau syndrome. The filled squares and circles indicate affected individuals. Arrow indicates the proband. The asterisks indicate the individuals who had undergone clinical and molecular genetic analyses in the study.

The study was approved by West China Hospital, Sichuan University Institute Review Board, and performed according to the principles of the Declaration of Helsinki. Informed consent was obtained from all participants. Three patients and four unaffected individuals, with age from 16 to 68 years old, were enrolled in this study. No consanguineous marriage was noticed in the family (Figure 1).

### Clinical examination

All individuals underwent ophthalmological examinations, including Snellen best-corrected visual acuity, detailed slit-lamp examination, and B-scan Ophthalmic Ultrasound. Blood tests for autoantibody to nuclear antigen (ANA), anti-streptolysin O (ASO), rheumatoid factor (RF), human leucocyte antigen-27 (HLA-27), C-reactive protein (CRP), and erydlrocyte erythrocyte sedimentation rate (ESR) were performed. X-rays of joints and chest and computed tomography (CT) scans were performed in all patients.

### Molecular genetic analysis

Peripheral blood was collected for DNA analysis from three patients and four unaffected individuals involved in this study. Genomic DNA was isolated from leukocytes using QIAamp DNA Blood Mini Kit (Qiagen, Hilden, Germany) by standard protocols. Coding sequences of *NOD2/CARD15* (exons 1–12, GenBank NG_007508.1) were amplified from genomic DNA using the forward and reverse primers shown in Table 1. Each 30 µl PCR amplification reaction was performed with 30–40 ng genomic DNA, 1.0 μM of each of the forward and reverse primers and 15 μl of 2× Taq Master Mix (SinoBio Biltech Co. Ltd, Shanghai, China). The modified thermal profile consisted of an initial denaturation step at 94 °C for 5 min, followed by a 35 cycles of denaturation at 94 °C for 30s, then annealing at 55 °C for 30 s and extension at 72 °C for 30 s, and a final extension at 72 °C for 5 min. The amplified products were purified with a cycle-pure kit (OMEGA; BioTek, Doraville, GA) and sequenced on the ABI 3730XL automated DNA sequencer (Applied Biosystems, Foster City, CA). Sequence data were compared pair-wise with wild type *NOD2* sequence (GenBank NG_007508.1).

**Table 1:** Primers used in polymerase chain reaction for amplification of *NOD2/CARD15*.

**Exon**	**Annealing temperature (°C)**	**Primer direction**	**Sequence (5′→3′)**	**Amplicon size (bp)**
1	55.0	Forward	GTAGACAGATCCAGGCTCAC	384
		Reverse	ACCAGCCAAGGATGCGACAG	
2	55.0	Forward	TGACCACCCTGCATCTGGCTT	627
		Reverse	ACCAAGTTACCCCACAGGCTG	
3	55.0	Forward	CAGTAAGCCTTCCCACATTG	451
		Reverse	AACACCTTTAGTTAGCCCTCA	
4a	55.0	Forward	CTGGCTCTCCTATCCCTTCA	555
		Reverse	TGTCTTCTTGACCAACATCAG	
4b	55.0	Forward	TCTCTTTGTCTTCCCATTCAG	462
		Reverse	AGGGCTGAGGTCTCTTGGA	
4c	55.0	Forward	GCTTCTCTGAACAGGGCATC	689
		Reverse	TGCTGTGATCTGAAGGTTGTG	
4d	55.0	Forward	AGGTGTCGTGCCAGGGAGTA	755
		Reverse	CACACTTAGCCTTGATGGTG	
5,6	55.0	Forward	GCACAGATGCTGGCACTTC	565
		Reverse	CAGATCAGACTGACTCAGGAAT	
7	55.0	Forward	GTAAACTAGACCTAGCAGCGA	277
		Reverse	CTCCATGCAGGTCCCTCTTC	
8	55.0	Forward	GGAGGAGGACTGTTAGTTCAT	362
		Reverse	AGAGGACAAGGGACATTTCCA	
9	55.0	Forward	AGACCAGGAGAGCACCACGA	301
		Reverse	CAGTCAATCACTCAATCATCCA	
10	55.0	Forward	TGTGAGTTCATCATCTTCCATA	297
		Reverse	ATCCTTGTCCACCTAGACCA	
11	55.0	Forward	CTCATTGGGAATCTCAGACAT	391
		Reverse	CAGAGAATCAGATCCTTCACAT	
12	55.0	Forward	GAGAGTCAGCCCATCCCAG	476
		Reverse	AGCAGAGGCCAGTCCCATACT	

The mutation was named according to the nomenclature recommended by the Human Genomic Variation Society (HGVS).

## Results

### Clinical findings

**Figure 2:**
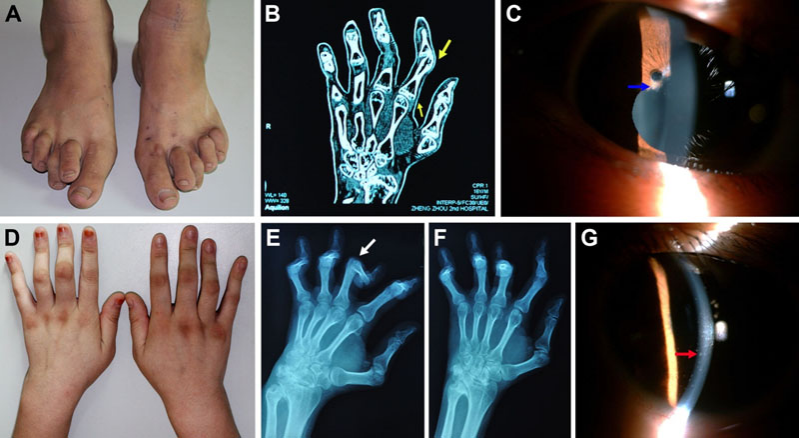
Representative photographs of arthritis and uveitis of patients in the family. **A**: General appearance showed deformities of the feet in patient (III:6). **B**: The coronal reconstruction section of CT scan of patient (III:6) revealed erosion of bone at the edges of the joint in the MCP joints, joint space narrowing and subluxation in the proximal interphalangeal (PIP) joints (yellow arrow). **C**: The right eye of the patient (III:6) revealed partial posterior synechia (blue arrow). **D**: Deformity of the hands of the proband (III:5). **E** and **F**: X-rays of patient (II:5) showed multiple and symmetric joint involvement, generalized osteoporosis, joint space narrowing, poorly defined edges of the articular surfer, subluxation, contracture and ankylosis of the PIP joints (white arrow). **G**: the right eye of the proband (III:5) showed dot-like calcific keratopathy in Bowman's membrane and the cornea between the opacities remained clear (red arrow).

The proband (III:5, Figure 1), a 20-year-old female, presented with a cutaneous eruption of discrete small papules when she was 3 years old, which was noticed by her mother. After several months, the skin rash faded away spontaneously without therapy, but subsequently recurred and persisted for months. At the age of 4, she had arthritis with periarticular swelling of metacarpophalangeal (MCP) and proximal interphalangeal (PIP) joints with no function problems. A few years later, she was diagnosed with juvenile rheumatoid arthritis (JRA), and treated with non-steroidal anti-inﬂammatory drugs (NSAIDs) initially and later, with intermittent courses of prednisone. Figure 2D revealed the deformities of the proband’s hands. At the age of 13, she had bilateral anterior uveitis and was treated with different dosages of topical corticosteroids, NSAIDs and immunosuppressive agents Cyclosporin A, which seemed to be ineffective in the treatment of uveitis. Secondary closed-angle glaucoma was found on her left eye when she was 15 years old. Trabeculectomy was then performed and intraocular pressure (IOP) was well controlled. Two years later after the operation, IOP began to rise up to about 40 mmHg on her right eye. Carteolol and brinzolamide were tried but failed to control IOP. She underwent peripheral iridectomy on the right eye. Now IOPs were well controlled in both eyes without any antiglaucoma medications. The uveitis progressed to the persistent fulminant panuveitis. The present visual acuity was CF (count fingers) OD and 20/400 OS. Anterior segment examination demonstrated bilateral band-shaped degeneration of cornea near the limbus and dot-like calcific keratopathy in Bowman's membrane (Figure 2G). The slit-lamp examination showed extensive posterior synechia of the iris (which caused occlusion of pupil) and opacity in the lens on her both eyes. Bilateral extensive goniosynechia and closed angles were showed under gonioscopy. B-scan Ophthalmic Ultrasound showed scattered flocculent or dot echo in the vitreous. Blood tests for autoantibody to nuclear antigen (ANA), anti-streptolysin O (ASO), rheumatoid factor (RF), human leucocyte antigen-27 (HLA-27) and C-reactive protein (CRP) were negative, and erythrocyte sedimentation rate (ESR) was within normal limits. X-rays and CT scans revealed periarticular osteoporosis and erosion of bone at the edges of the joint. The joint space became narrow and subluxation at some MCP and PIP joints, while Chest X-ray was normal.

The proband’s brother (III:6, Figure 1), a 16-year-old male, presented with periarticular swelling of the wrists, MCP and proximal PIP joints followed by exanthema on the face, trunk and extremities at the age of 4. During the course of the disease, the patient got recurrent episodes of skin rashs on the face and extremities. Figure 2A showed the deformities of the MCP and PIP joints. He had mild anterior uveitis at the age of 13. Thesymptoms and signs were well controlled with low doses of prednisone and NSAIDs. His current best corrected visual acuity was 20/25 OD and 20/20 OS. Slit-lamp examination revealed partial posterior synechia from 2:30 to 3:00 and 9:30 to 10:30 of the iris on his right eye (Figure 2C). His blood tests for ANA, ASO, RF, HLA-27, and CRP were also negative, ESR was within normal limits. The coronal reconstruction section of CT scans showed erosion of bone at the edges of the joint in the MCP joints andjoint space narrowing and subluxation in the PIP joints (Figure 2B). Chest X-ray was normal. The diagnosis was juvenile rheumatoid arthritis then.

The proband’s father (II:5, Figure 1), a 43-year-old male, presented with MCP and PIP joint changes resembling their children at the age of about 6 but not manifested with exanthema. He also had slight anterior uveitis at the age of 25, and his best corrected visual acuity was 20/20 OD and 20/20 OS. The ocular manifestation was partial posterior synechia similar to his son. His blood tests for ANA, ASO, RF, HLA-27, and CRP were negative, ESR was within normal limits. Chest X-ray was also normal. X-rays of his both hands showed multiple and symmetric joint involvement, generalized osteoporosis, joint space narrowing, poorly defined edges of the articular surfer, subluxation, contracture and ankylosis of the PIP joints (Figure 2E,F).

### *NOD2/CARD15* analysis

**Figure 3:**
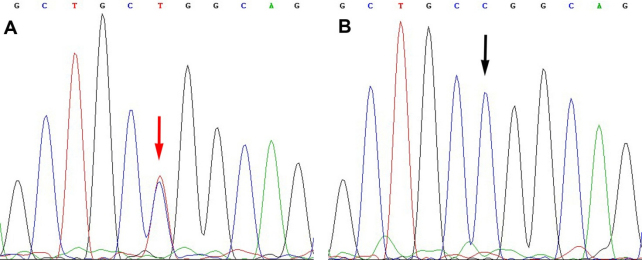
*NOD2/CARD15* heterozygous mutation in the family. **A**: A heterozygous mutation consisting of a C>T transversion in exon 4 (red arrow) was identified. **B**: Wild type sequence from an unaffected member (black arrow).

All exons of *NOD2/CARD15* of the affected and unaffected individuals included in this study were analyzed by direct sequencing. A heterozygous mutation C>T transversion in exon 4 was identified in each patient, which resulted in an amino acid substitution from Arginine (CGG) to Tryptophan (TGG) at codon 334 (R334W). No such mutation was found in unaffected members who were taken as controls in the family (Figure 3).

## Discussion

Sarcoidosis is a systemic inflammatory disease characterized by swelling of the bilateral hilar lymph nodes and the presence of noncaseating epithelioid cell granulomas. As a special subtype of sarcoidosis, early-onset sarcoidosis (EOS) occurs in children younger than 4 years of age and is characterized by a distinct triad of skin, joint, and eye disorders [14]. Blau syndrome (BS), which demonstrates similar clinical manifestations as EOS, has been described as a fixed triad of multiorgan granulomatous arthritis, uveitis and dermatitis with a clear dominant inheritance pattern [1]. Since 1996 the BS locus was mapped to chromosomal 16p12-q21 [15] and NOD*2/CARD15* was subsequently identified in that region to be associated with the disease [6], more than 14 *NOD2/CARD15* mutations have been described in many different countries, and have been shown to affect the residue Arg334, which may be a genetic “hot spot” for mutations in patients with some familial granulomatoses [6,12,16,17]. Previous studies showed BS shared considerable pathologic, clinical and therapeutic overlap with sarcoidosis, but no subject of BS has yet manifested sarcoidlike lung disease [18,19]. Other granulomatous diseases such as Wegener’s granulomatosis and ordinary sarcoidosis have not been associated with *NOD2/CARD15* [20-23], while the affected individuals with EOS which also have a *NOD2/CARD15* mutation were always sporadic cases without family history of the disease [14].

Besides the symptoms of arthritis, uveitis and dermatitis, some atypical clinical features such as central nervous system involvement, interstitial pneumonitis, liver involvement, sinus of valsalva aneurysm, periodic fever, and leg ulcers were also reported during the course of the disease [24-29]. In this study, the pedigree is inherited as an obvious autosomal dominant trait with early onset below the age of 4. The two patients manifested typical granulomatous arthritis, anterior uveitis and skin rash of BS, while the proband’s father presented with both arthritis and uveitis but no manifestation of dermatitis. The changes in joints were noticed in the early age of onset of all patients. None of the patients had lung and other organs involvement. The female proband, compared with her father and brother, presented with more severe anterior uveitis companied by glaucoma and cataract. Besides, the age of disease onset was later in the father than in the son and daughter. This suggests a possible case of genetic anticipation, which has been reported in several BS families [12,17,30,31]. From a clinical point of view, the presence of multiple atypical manifestations, the occurrence of incomplete forms of the classic triad, and the varying degrees of severity from case to case confirmed the presence of a degree of clinical heterogeneity in BS, even in patients from the same family [17,32]. Interestingly, some studies suggested that BCG (Bacillus Calmette-Guerin) vaccination and other microorganisms may be an obvious triggering factor in the ocular and joint inflammation seen in BS [33-35]. The case herein reported further demonstrates the heterogeneity of BS that should be taken into account when genetic screening is performed in those cases with atypical presentation.

Almost all the patients in this family were originally misdiagnosed as juvenile rheumatoid arthritis (JRA). JRA had also been reported in other patients with BS [2,12,36], the main reason for the incorrect diagnosis could be the high prevalence of JAR and the similar clinical features of the joints. In this pedigree, mutational analysis of *NOD2/CARD15* revealed a heterozygous mutation (R334W) previously detected in other Blau syndrome pedigrees in different ethnic nationalities and countries [6,37,38]. It is considerately significant to distinguish this genetic disease from other pediatric arthritis, such as juvenile rheumatoid arthritis, juvenile ankylosingspondylitis and EOS not only for treatment but also for genetic counseling and prenatal diagnosis. Until now, no asymptomatic carriage has been reported, so molecular genetic analysis rather than tissue sampling may prove to be the most efficient way to make an accurate diagnosis [39].

Despite the causative genes have been identified, the molecule mechanism and the pathogenesis of the disorder is not fully understood. *NOD2/CARD15* is a member of the Nod1/Apaf-1 family, It encodes a cytosolic protein termed nucleotide oligomerization domain 2 (Nod2), comprising two caspase recruitment domains (CARD): a nucleotide oligomerisation domain (NOD), and a stretch of leucine rich repeats (LRRs) [40]. The Nod2 protein, a cytosolic receptor which belongs to the group of Nod-like receptors (NLRs) plays an important role in the immune response against bacterial peptidoglycan by inducing signaling pathways such as NF-kB (nuclear factor kappaB) and MAPKs (mitogen-activated protein kinases) [41]. Constitutive NF-kB activation was found in majority of EOS and BS cases which may share the common genetic etiology of *NOD2/CARD15* [42]. Mutations of the NOD and LRR seem to result in different phenotypic consequences within the spectrum of granulomatous disorders. For example, the Crohn's disease-associated CARD15 mutations are located in or close to the LRRs region, while Blau syndrome mutations were found to cluster in the NOD [20,43]. McDonald et al. suggested Erbin, a protein involved in cell polarity, receptor localization, and regulation of the mitogen-activated protein kinase pathway, as a regulator of Nod2 signaling [44].

Collectively, our results demonstrate that the R334W mutation of *NOD2/CARD15* caused BS in a Chinese pedigree. Molecular genetic analysis is necessary to establish the appropriate diagnosis of BS. This is the first report of an R334W mutation in *NOD2/CARD15* in a Chinese population with BS. Obviously, more studies of the molecule pathomechanisms of BS are needed in further investigations.
